# Proof of principle study of ultrasonic particle manipulation by a circular array device

**DOI:** 10.1098/rspa.2012.0232

**Published:** 2012-07-11

**Authors:** Alon Grinenko, Paul D. Wilcox, Charles R. P. Courtney, Bruce W. Drinkwater

**Affiliations:** Department of Mechanical Engineering, University of Bristol, Bristol BS8 1TR, UK

**Keywords:** ultrasonics, acoustic radiation force, particle manipulation

## Abstract

A feasibility study of a circular ultrasonic array device for acoustic particle manipulation is presented. A general approach based on Green's function is developed to analyse the underlying properties of a circular acoustic array. It allows the size of a controllable device area as a function of the number of array elements to be established and the array excitation required to produce a desired field distribution to be determined. A set of quantitative parameters characterizing the complexity of the pressure landscape is suggested, and relation to the number of array elements is found. Next, a finite-element model of a physically realizable circular piezo-acoustic array device is employed to demonstrate that the trapping capability can be achieved in practice.

## 1. Introduction

The acoustic radiation force (King 1934) is the basis of ultrasonic methods of particle manipulation. The technique is complementary to optical and dielectric methods, in particular for larger biological cells (Hultström *et al.* 2007; Bazou *et al.* 2008) where optical methods reach their limits, and for particles that do not posses high optical contrast in a medium. Owing to the typical micrometre scale of the fields and nanoNewton force magnitudes, ultrasonic particle manipulation techniques are used in applications where large numbers of particles need to be handled, as in particle fractionation (Yasuda *et al.* 1995; Ratier & Hoyos 2010), sorting (Johnson & Feke 1995) and medium exchange (Augustsson *et al.* 2009) devices or for cell agglomeration that allows filtering of cells from fluids (Coakley 1997; Coakley *et al.* 2000). Also, the acoustic radiation force was reported to be used in the production of meta-materials (Saito *et al.* 1998, 1999; Mitri *et al.* 2011).

Of high interest are more general-purpose acoustic devices that allow particles to be manipulated around the acoustic cavity rather than driven to specified locations determined by a stationary acoustic field. A review in Courtney *et al.* (2012) indicates four types of acoustic particle manipulation approaches. These are: mode switching, ‘acoustical tweezers’, linear arrays and counter-propagating waves.

The mode switching manipulation method is based on trapping the particles at the nodes of resonant standing waves and spatially shifting the nodes by means of switching between resonant frequencies (Trinh *et al.* 1986; Min *et al.* 1992; Glynne-Jones *et al.* 2010). The acoustic tweezers method is similar in principle to the optical tweezer technique (Ashkin *et al.* 1987) and operates by trapping the particles in the focal point of an acoustic beam (Wu & Du 1990; Wu 1991; Lee & Shung 2006; Lee *et al.* 2009). Particle manipulation in this method is achieved by physical displacement of the transducers generating the field. A development of this approach based on manipulation by propagating Bessel beams was analysed in the work of Marston (2006) and Mitri (2008). In the method based on linear arrays, two-dimensional traps are formed by activating selected elements of the array opposing a passive reflector (Kozuka *et al.* 1998; Démoré *et al.* 2010). Here, the manipulation is achieved by selective switching of the transducers. The last approach involves generating a standing wave as a sum of two counter-propagating waves. This allows the locations of the field nodes to be changed by varying the relative phase between the two sources. The problem of generating counter-propagating standing waves without generating resonant standing waves (Kwiatkowski & Marston 1998) was resolved by using transducers acoustically matched to the fluid with an absorbing backing to prevent reflections leading to resonant modes (Courtney *et al.* 2010). This method allows a manipulation of particles on a two-dimensional plane to be achieved using a pair of orthogonal phase-controlled counter-propagating waves (Courtney *et al.* 2012).

**Figure 1.**
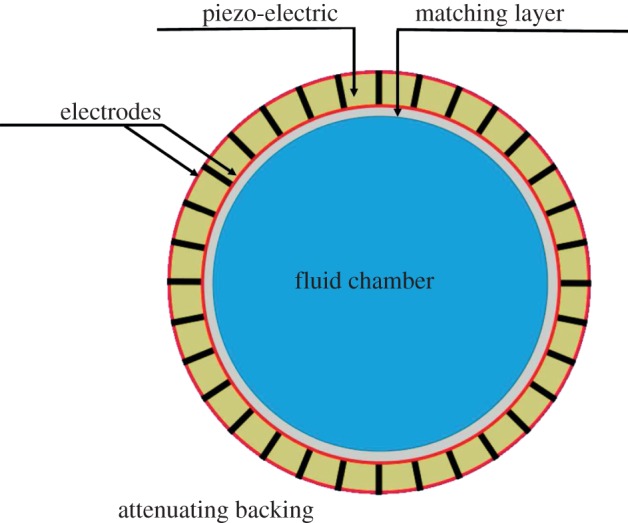
Schematic of a 16 element circular piezo-electric transducer array device. Each element in the array is excited by an individual voltage source. Matching between the transducers and the fluid is achieved using a quarter-wavelength matching layer and the attenuating backing is used to absorb the waves reflected from the back face of the transducers. (Online version in colour.)

The last approach allows multiple particle traps located at the nodes of a two-dimensional lattice to be moved en masse but does not allow the independent control of individual traps. Greater flexibility is therefore required to match the particle manipulation dexterity achieved using the optical tweezers (Ashkin *et al.* 1987). Thus, the principles of the device design reported by Courtney *et al.* (2012) are taken a step forward to produce a dextrous acoustic manipulation system. It is based on a circular ultrasonic array such as shown in figure 1. This conceptual device consists of a piezo-ceramic ring divided into a number of identical separated elements, an absorbing backing layer, a matching layer and a fluid chamber. The matching layer is used to optimize the coupling of the acoustic signal into the fluid chamber in which the particle trapping and manipulation take place. To generate an acoustic radiation force landscape **f** (King 1934) related to acoustic force potential *U* (Gorkov 1962) as **f**=−**∇***U*, each transducer in the array is individually excited through a dedicated control channel. The device excitation is designed to create a force potential landscape with wells in which particles can be trapped, where the force potential *U* on a spherical non-elastic particle is given by
1.1
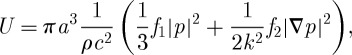
where *p* is the total pressure field, *a* is the radius of the particle, *ρ* is the medium density, *c* is the speed of sound and the coefficients *f*_1_ and *f*_2_ are defined as
1.2
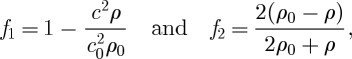
where *ρ*_0_ and *c*_0_ are the density and the speed of sound in the particle, respectively (Gorkov 1962). Because the force potential field in equation (1.1) created by a pressure distribution *J*_*α*_(*r*), where *J*_*α*_(*r*) is a Bessel function of the first kind of order *α*, produces a potential with a local minimum at *r*=0 for orders *α*>0 (Marston 2006; Mitri 2008), such pressure distribution is capable of trapping particles with densities *ρ*_0_>*ρ* and would be a natural choice of the physically viable field in a circular geometry characteristic of the device considered here. For particles with small densities *ρ*_0_<*ρ*, a *J*_0_(*r*) field similar to the optical tweezers (Ashkin *et al.* 1987) should be considered.

The purpose of the analysis presented in the manuscript was to demonstrate the feasibility of the circular ultrasonic array device to form and independently manipulate multiple acoustic radiation force traps for dextrous particle manipulation.

The analysis of the device is divided into two parts. In the first part, fundamental properties of a generic circular acoustic array device are examined. Here, the range of acoustic traps obtainable in the device is found, the dependance between the fraction of the device area in which control over the acoustic field can be achieved and the number of array elements is established, and the relation between the field at the boundary and the field inside the area enclosed by the boundary is determined. This relation allows the required excitation of the array elements in a practical device to be calculated and the complexity of the radiation force landscape to be characterized by a set of quantitative parameters. The maximum complexity attainable for a given number of array elements is found.

In the next part of the analysis, these general properties are applied to the more realistic device shown in figure 1. The major difference from the first part of the analysis is the model of the source of acoustic radiation at the device boundary. Whereas in the first part of the analysis, the boundary is formed by an array of non-reflecting matched sources, in the realistic device, the piezo-elements forming the boundary are mutually reflective because the transducer-to-fluid matching is not perfect. Therefore, finite-element (FE) modelling is required in the latter case to establish the acoustic field generated by an excited array element. This field, together with the relation between the fields on the boundary and in the area enclosed established in the previous part, is used to determine the excitation of the array that produces the required trapping field.

## 2. General formalism

### (a) Green's function approach

Control over the field distribution in the fluid is achieved by imposing the field on the fluid boundary using a transducer array. The required field on the boundary can be found using a reciprocity relation by starting with a physically viable field in the volume enclosed, and tracing it back to the boundary. However, the reciprocity cannot be implied straightforwardly for a discontinuous and open boundary. In the following discussion, an analysis is presented to establish the relation between the boundary and volume fields in such cases.

In general, the relation of the fields on the boundary and in the fluid volume enclosed can be found using an integral representation derived from Green's theorem for steady-state harmonic waves (Morse & Feshbach 1953, p. 803). For a fluid volume with no body forces, the pressure at an internal point *P*∈*V* can be written as (Morse & Feshbach 1953, p. 806)
2.1
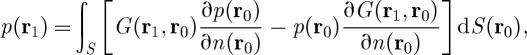
where *G*(**r**_1_,**r**_0_) is Green's function and *n*(**r**_0_) is the coordinate in the direction of the normal on a boundary *S*. This integral representation is not directly applicable because to calculate the pressure at an internal point, both the pressure *p* and its normal derivative on the boundary must be known. However, taking *G*(**r**_1_,**r**_0_) as a combination of fundamental solution of the Helmholtz equation due to a point source −*δ*(**r**_1_−**r**_0_) inside the volume *V* and a homogeneous solution, one of the terms in equation (2.1) can be dropped (Schmerr 1998, pp. 157–159).

**Figure 2.**
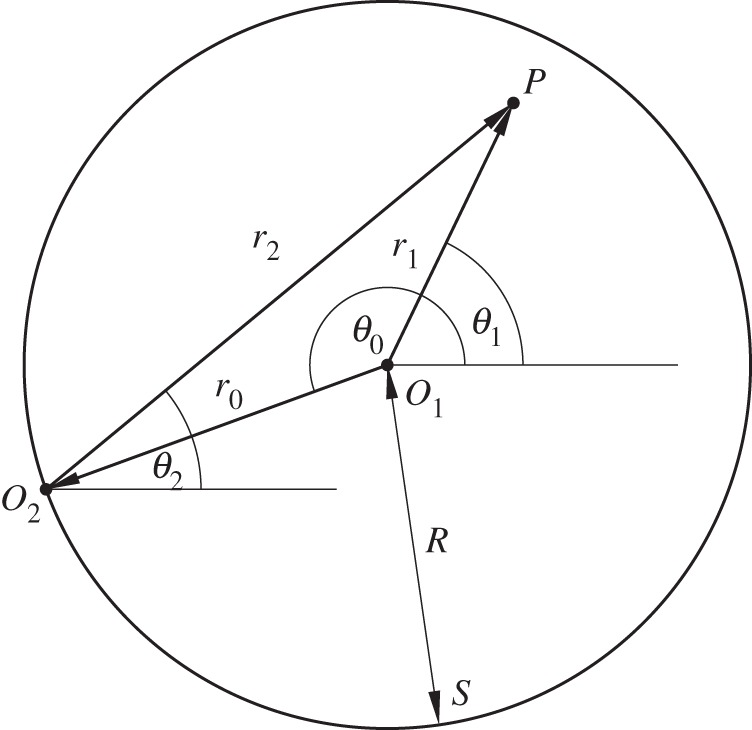
Definition of coordinate systems used in the integral representation in equation (2.1).

The circular array device is described in two-dimensional plane by the coordinate system shown in figure 2, where **r**_0_=(*r*_0_,*θ*_0_) and **r**_1_=(*r*_1_,*θ*_1_) are defined in accordance with equation (2.1) as polar coordinates of the internal point *P* and the point source relative to the common origin *O*_1_, whereas **r**_2_=(*r*_2_,*θ*_2_) is the polar coordinate of an internal point *P* with respect to the origin *O*_2_ located on boundary *S*. The boundary *S* in equation (2.1) is defined by a circle of a fixed radius *r*_0_=*R* and the corresponding volume *V* enclosed by this circular boundary is an infinitely long cylinder of radius *R*. The fundamental solution of the Helmholtz equation in cylindrical coordinates due to a harmonic line source of wavelength *λ* located at **r**_0_ (figure 2) can be written using Graff's addition theorem as (Martin 2006, pp. 29–61) follows:
2.2
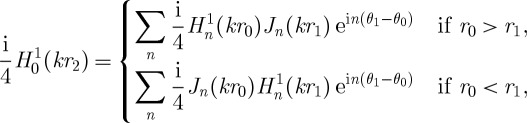
where the wavenumber *k*=2*π*/*λ*. Green's function satisfying the boundary condition *G*(**r**_1_,**r**_0_)_*r*_0_=*R*_=0 is
2.3
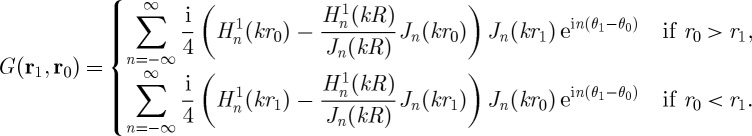
With this choice of Green's function, the first term in equation (2.1) vanishes and the pressure inside the volume *p*(**r**_1_) is uniquely determined by the pressure on the boundary *p*(*R*,*θ*_0_),
2.4
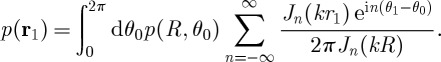

The relation of the boundary and volume pressures implied by equation (2.4) is just an expression of the uniqueness theorem for the Helmholtz equation, stating that for a given boundary condition, the solution inside the volume enclosed is uniquely determined. Practically, this implies that by controlling the pressure distribution on a boundary, any pressure distribution satisfying the Helmholtz equation can be generated inside the volume enclosed. In the case of single-frequency devices, the acoustic landscape is constructed from linear combinations of Bessel functions of the first kind, which are the physically viable non-divergent solutions of the Helmholtz equation. Bessel function traps *p*(**r**)∝*J*_*α*_(*r*) e^i*αθ*^, of orders *α*>0 will be considered in the following because only such traps generate a trapping field at the centre *r*=0 for particles that are denser than the fluid (Marston 2006; Mitri 2008).

**Figure 3.**
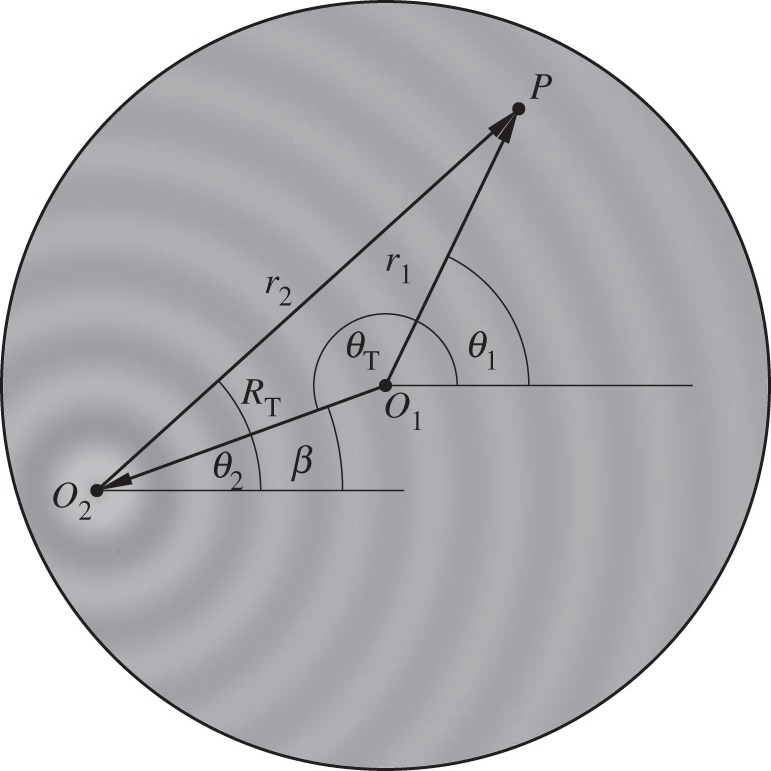
Definition of coordinate systems used in the Graff's transformation equation (2.5) with a prototypical *J*_1_(*k***r**_2_) trap located at (*R*_T_,*θ*_T_).

A Bessel pressure distribution of order *α* centred at a point **r**_0_ with *r*_0_=*R*_T_ and *θ*_0_=*θ*_T_ (figure 3) can be expressed in terms of **r**_1_ using Graff's addition theorem as
2.5
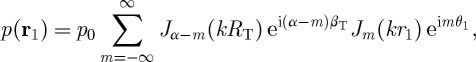
where *β*_T_=*θ*_T_−*π* and *p*_0_ is the pressure amplitude. The corresponding pressure on the boundary is
2.6
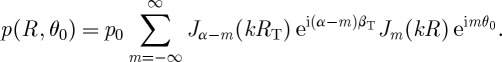
The fact that this boundary pressure gives exactly the pressure distribution as in equation (2.5) can be readily verified by substituting the last expression into the integral in equation (2.4)
2.7
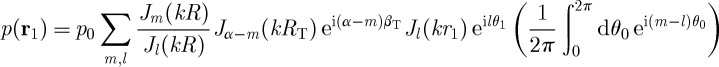
and using
2.8
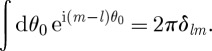
A practically important case of a discontinuous boundary will be considered next.

### (b) The effects of a discontinuous boundary and the number of array elements

In the circular array device analysed here, the fluid boundary is controlled by a periodic array of separate piezo-elements. The effect of replacing the continuous circular boundary by a finite number *N* of sources equally distributed along the boundary can be qualitatively understood using the Nyquist theorem. Assuming a linear array and substituting the spacing of *D*=2*πR*/*N* between the elements into the Nyquist condition 1/*D*>2/*λ* gives the minimum number of elements to achieve a non-aliased signal *N*_min_=4*πR*/*λ*. If this requirement is violated, aliasing will occur at large angles relative to the normal to the inner surface of the boundary, i.e. in a ring 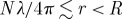 adjacent to the inner boundary surface. To evaluate the effect of replacing the continuous boundary by a finite number *N* of sources in a circular array quantitatively, we start by representing the boundary by a piecewise constant function of constant pressure on segments of Δ*φ*<Δ length, where Δ=2*π*/*N*, and with gaps of zero pressure in between. The integral in equation (2.7) is then replaced by the sum
2.9
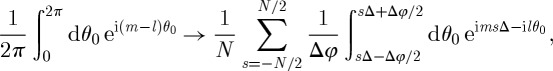
which, using the Poisson summation formula, can be expressed as

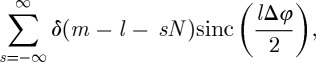
and the pressure in the volume becomes
2.10
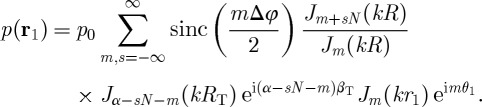
To further simplify the analysis, we consider the case of infinitely small sources 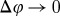 such that 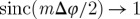. The practical validity of this assumption is demonstrated towards the end of this section.

The sum in the last equation can be broken into 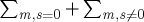, where the *s*=0 part gives the principal term *p*_T_(**r**_1_)=*p*_0_*J*_*α*_(*k***r**_2_) e^i*αθ*_2_^ and the remainder contributes to the artefact field 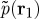 created owing to the boundary discontinuity. To determine the artefact field
2.11
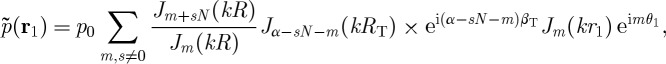
we consider the asymptotic behaviour of the Bessel function for large orders (Abramowitz & Stegun 1964, p. 365),
2.12
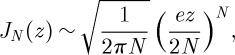
from which one can conclude that while the first term in equation (2.11), *J*_*m*−*N*_(*kR*)/*J*_*m*_(*kR*), is limited for *m*<*πeR*/*λ*, the second term, *J*_*α*−*sN*−*m*_(*kR*_T_), becomes zero for |*m*+*sN*−*α*|>*πe*(*R*_T_/*λ*). Therefore, if there exist *m* such that *J*_*m*−*N*_(*kR*)/*J*_*m*_(*kR*) is limited ∀*m*: |*m*+*sN*−*α*|<*πe*(*R*_T_/*λ*), the artefact field 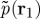 can be estimated by the sum over a finite number of orders of *m*,
2.13
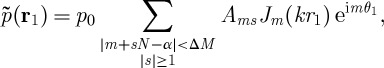
where Δ*M*=*πeR*_T_/*λ* and *A*_*ms*_ are the appropriate coefficients in equation (2.11).

When *R*_T_ increases, the largest order for non-zero *J*_*α*−*sN*−*m*_(*kR*_T_) (e.g. *N*+*α*+*πe*(*R*_T_/*λ*) for *s*=−1) becomes greater than *πeR*/*λ*. In this case, only the lower order terms in *m* can be neglected such that in general the artefact field can be approximated by
2.14
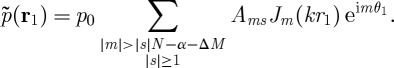


**Figure 4.**
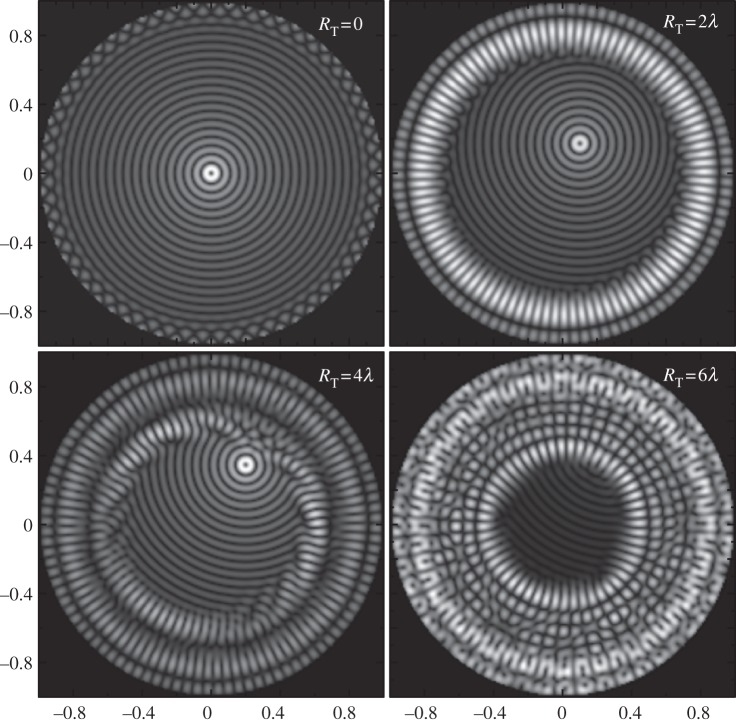
A *J*_1_ Bessel trap generated by a 60 element point-source array with *R*=10*λ*. In two of the cases shown, the trap centre distance from the centre of the array is *R*_T_<1/2*λ*(*N*/*πe*)∼3.5*λ* and in the other two cases, this condition is violated.

In order to understand the structure of equation (2.14), consider first the *R*_T_=0 case, in which the only contribution comes from *m*=±*N*+*α* terms. In this case,
2.15
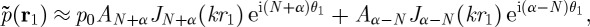
which, with the help of the asymptotic behaviour equation (2.12), indicates that the artefact field introduces a disturbance outside a circle of radius *r*_1_=*λ*(*N*−*α*)/*πe*. The disturbance field is determined by *J*_*N*±*α*_(*kr*_1_) and goes to zero for *r*_1_<*λ*(*N*−*α*)/*πe*. This case is depicted in figure 4, where *p*(**r**_1_) calculated using equation (2.7) for *α*=1, *N*=60 and a set of different values of *R*_T_ is shown. Because Δ*M* increases with *R*_T_, the lowest order *m* contributing to the artefact field 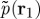 decreases as implied by equation (2.14). Thus, the disturbance-free region is reduced to *r*<*λ*(*N*−*α*)/*πe*−*R*_T_, as shown in figure 4.

The maximum value of *R*_T_ can be found by demanding that the trap lies inside the distortion-free region. This requirement implies that the useful area of the fluid chamber is defined by
2.16
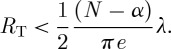
In the example shown in figure 4, the maximum value of *R*_T_ corresponding to *N*=60 and *R*=10*λ* is *R*_T_∼3.5*λ*. The plots of *p*(**r**_1_), calculated according to equation (2.4), show that indeed, for *R*_T_<3.5*λ*, the trap field appears undistorted; for *R*_T_=4*λ*, the trap starts to overlap with the artefact field, and for *R*_T_=6*λ*, the trap is completely destroyed in the artefact field. This set of plots also illustrates how the undistorted region decreases with increasing *R*_T_.

Reversing the argument used to obtain the condition on the maximum useful region in equation (2.14), the minimum number of elements such that the useful region is at least as large as the inner radius of the fluid chamber *R* can be found to be
2.17
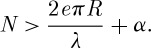
This is a more stringent condition than the well-known Nyquist sampling condition *N*>4*πR*/*λ* on account of the cylindrical geometry of the device.

Up to this point in the analysis, the effect of the finite source size Δ*φ*>0 has been neglected. This assumption is justified if *sinc*(*m*Δ*φ*/2) varies slowly for 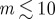. From here, the maximum size of the source can be estimated as Δ*l*∼*πR*/20, which is readily satisfied if the number of sources is *N*>40.

This section can be summarized by indicating that as a result of a boundary discontinuity, an artefact field appears in addition to the principal trapping field. Because the artefact field is negligibly small inside the circular area defined by equation (2.14), it affects the principle field only outside this circle, whereas the field inside remains virtually undisturbed. This means that potentially any pressure field satisfying the Helmholtz equation can be generated by an array of *N* elements within the area defined by equation (2.14), and the finite number of array elements limits only the spatial extent of the controllable area but not the complexity of the pressure landscape. The complexity of the landscape is determined by the interaction of pressure fields of individual traps, which, in the case of a single operating frequency device considered here, are given by Bessel functions.

### (c) Multiple trapping

Selective manipulation of multiple particles is an essential capability of the device analysed here for which an ability to generate complex force landscapes with separated particle traps is required. The trapping complexity grows with the number of traps *N*_p_ introduced; thus, given the number of traps *N*_p_, the minimum average spacing between the traps *d*_min_ is established to characterize the configuration complexity. For example, it is clear that for two particles, the minimum spacing will be *λ*, because at closer distance, two separate traps will blur into one.

**Figure 5.**
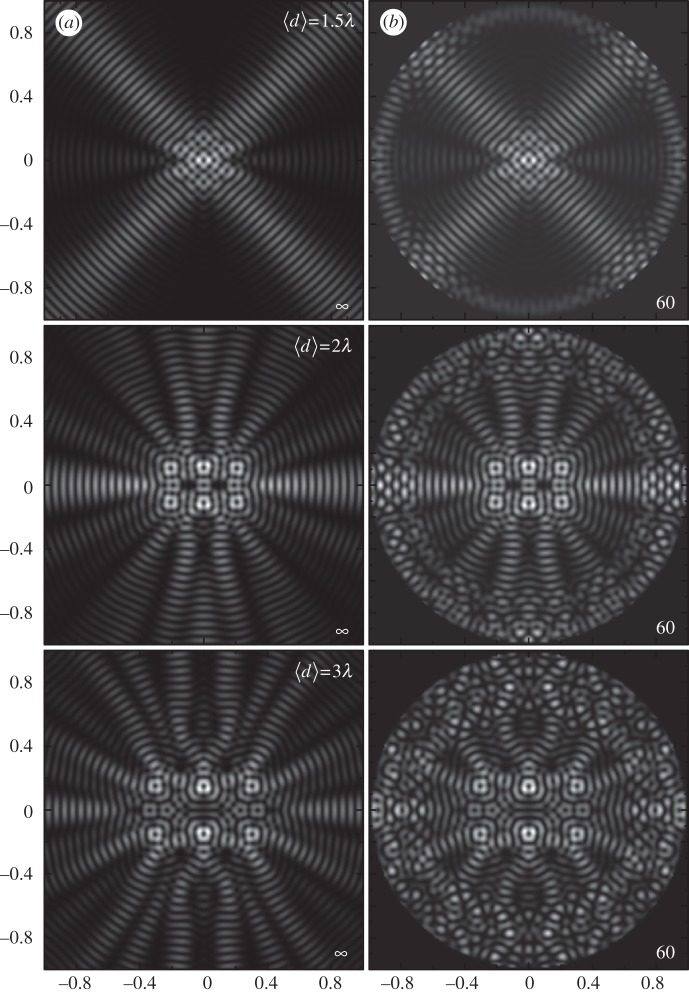
Force potential *U* for a polystyrene bead with six traps generated by (*b*) a 60 element point-source array and (*a*) a continuous boundary. Potential landscapes with different average trap spacing *d* are shown.

The average spacing between the particles is defined using the area *S* occupied by the particles as
2.18
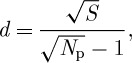
where *S* is the area of the smallest rectangle enclosing all the particles, and *d*_min_ is defined as the smallest spacing *d* between the traps for which the force potential *U* contains *N*_p_ individual and separate potential wells that allow *N*_p_ particles to be trapped. This loose definition of *d*_min_ is better explained graphically. In figure 5, a potential landscape *U* given for configurations of *N*_p_=6 traps with different average spacings *d* is shown. Clearly, for *d*=1.5*λ*, the trapping ability of the field is absent but appears for the spacing of *d*=2.0*λ*; in that case, six separate closed traps become apparent. Thus, *d*_min_=2.0*λ* for *N*_p_=6 configuration.

The parameter *d*_min_ is independent of the number of array elements *N*. This can be further seen from figure 5. The plots shown in figure 5*a* correspond to an ideal case with a continuous boundary, and in figure 5*b*, the same configurations are obtained with a 60 element point-source array. One can see that in the case of an array with a finite number of elements, the field inside the circle defined by equation (2.14) is identical to the field generated by a continuous boundary. This indicates that *d*_min_ is independent of *N*; however, the maximum spatial extent of the configuration is limited according to the condition in equation (2.14).

Heuristically determined minimum average spacing *d*_min_, minimum area *S*_min_, minimum extent *R*_min_ and the minimum number of array elements *N*_min_ required are shown in table 1 for different *N*_p_. For example, for a configuration with *N*_p_=6 traps, a minimum of *N*_min_=48 array elements are required to contain the area of *S*_min_=8*λ*^2^ lying within a circular controllable area of radius *R*_min_=2*λ*, where *N*_min_ is found by substituting *R*_min_ into equation (2.14). Thus, any of the parameters listed in the table can be regarded as a function characteristic of the configuration complexity with a variable *N*_p_. Also, one can see that these functions grow as a function of *N*_p_. For estimation of the complexity, an approximate formula for the minimum extent *R*_min_ can be used,
2.19
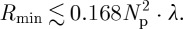

**Table 1.** The minimum average spacing *d*_min_, the minimum area *S*_min_ of the configuration, the minimum lateral extent of the configuration *R*_min_ and the minimum number of array elements required for generation of the configuration *N*_min_ as a function of trap number *N*_p_.

*N*_p_	*d*_min_	*S*_min_	*R*_min_	*N*_min_
2	*λ*		0.5*λ*	9
4	*λ*	*λ*^2^	0.5 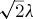	12
6	2*λ*	8*λ*^2^	2 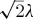	48
9	5*λ*	100*λ*^2^	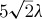	120
12	12.2*λ*	900*λ*^2^	15 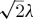	362
16	20*λ*	3600*λ*^2^	30 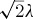	724

## 3. Finite-element model of the device

In §2, the underlying properties of a circular acoustic array were derived by assuming some pre-existing boundary conditions and by analysing their effect on the field in the volume enclosed. However, the question of how the required boundary conditions can be realized in practice was not addressed and is the subject of this section.

In a practical device such as the one shown in figure 1, the field in the fluid volume is produced by a piezo-ceramic transducer array. The field generated by each element in the array is evaluated using an FE model. This calculated field is then used to establish the excitation of the array elements required to produce a boundary condition such as determined in §2.

The FE model of the device was obtained using a commercial FE package (PZFlex; Weidlinger Associates Inc.). The device consists of a piezo-ceramic ring (PZ26, Ferroperm piezoceramics A/S) of inner radius 5.35 mm and wall thickness 1 mm, divided into 16 elements, separated by air-filled gaps and with a backing layer and a quarter wave (341 μm at 2 MHz) matching layer (15% by volume alumina-loaded epoxy).

A two-dimensional model, assuming plane strain in the perpendicular direction, was used and solved with a time-stepping algorithm. In order to reduce the size of the model, only half the system was modelled with a symmetrical boundary condition at the mid-plane *x*=0 and, rather than model a backing layer thick enough to absorb all incoming radiation, backing material (10% by volume tungsten-loaded epoxy) was modelled extending the modelled area out to a square of width 4 mm wider than the outer diameter of the piezo-electric array, and absorbing boundary conditions applied to remove reflections from the boundaries of the model. In a practical device (Courtney *et al.* 2012), the backing layer of 9 mm thickness was used, absorbing 99 per cent of the incident energy.

Since all the array elements are assumed to be identical, it is sufficient to calculate the system response to a single excited element, e.g. the bottom element in figure 1. This element was excited with a single cycle at 2 MHz and the model run until pressure in the device had decayed to negligible levels. Steady-state responses (in terms of pressure complex amplitudes) were calculated by performing Fourier transforms of the response at each node of the model, extracting the complex amplitude at 2 MHz and normalizing to the applied voltage amplitude at the corresponding frequency.

**Figure 6.**
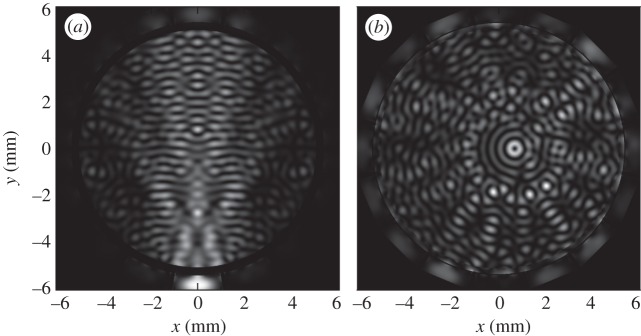
FE-based calculation of a force potential *U* in a 16 element device. (*a*) Single device excited at the bottom of the array. (*b*) All the elements excited to generate a trap at *R*_T_∼0.937*λ* distance from the centre.

Given the response of the system to excitation of one element (figure 6a), the response to excitation of multiple elements can be calculated as the linear superposition of the single-element response, rotated appropriately and scaled with a complex amplitude to allow different excitation amplitudes and phases to be applied to each transducer. The total field in the fluid chamber is thus given by
3.1
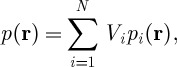
where *p*(**r**) is the total pressure in the fluid chamber, *p*_*i*_(**r**) is the pressure response of the system to the excitation of an element *i* and *V*_*i*_ is the excitation amplitude of the element.

According to §2, in order to generate the required pressure landscape *p*(**r**), it is sufficient and necessary to impose the corresponding boundary pressure *p*(*r*=*R*,*θ*) at some *R*. However, given only an *N* element device, it is only possible to satisfy this boundary condition approximately. To determine the optimal amplitudes (*V*_*i*_) giving the best approximation, the quadratic average error function between the required boundary pressure *p*(*R*,*θ*) and the boundary pressure generated is defined as
3.2
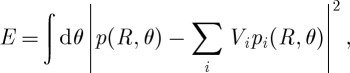
and the values of *V*_*i*_ are found by solving the set of 2×*N* linear equations derived from the condition
3.3
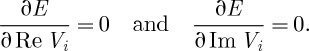
The force potential distribution with a trap at *R*_T_∼0.937*λ*, corresponding to the maximum displacement distance according to equation (2.11), obtained using the outlined optimization procedure, is shown in figure 6b. Here, similar to the idealized case analysed in §2, the field inside the area defined by equation (2.11) is distortion free, and the field outside this area is dominated by the artefact field contribution.

This analysis demonstrates that it is possible to impose the boundary conditions necessary for creating pressure traps using a physically realizable ultrasonic array (figure 1) based on the fields predicted by an FE model. However, it is clear that the successful operation of a physical device using this method depends on how accurately the FE model represents the performance of each array element. For example, a real device is three dimensional, there will be inter-element variability and there may be other manufacturing imperfections leading to deviations between a numerical model and the true performance. The robustness of the proposed method depends on its insensitivity to such deviations, which are likely to impact on the physical design of the device. This is the subject of ongoing research.

## 4. Conclusions

A circular transducer array device (figure 1) for manipulation of particles was analysed. The purpose of the analysis was to examine the ability of the device to produce pressure landscapes required for particle manipulation. This proof of principle analysis was carried out in two steps. First, a general theoretical approach based on Green's function integral representation of volume pressure as a function of the boundary pressure was developed. This formalism has been used to establish the upper limit of controllable area of the operational volume as a function of the number of array elements and to evaluate the complexity price function as a function of the number of traps. Using the conclusions drawn from the formal approach, an FE model of a practically realizable device was used to obtain a preliminary assessment of its feasibility. Although a further in-depth modelling of the practical device is essential, the preliminary results presented indicate the feasibility of using such a device for particle manipulation.
